# Systemic Manifestation of Periprosthetic Joint Infection Is Associated With Increased In-Hospital Mortality

**DOI:** 10.7759/cureus.36572

**Published:** 2023-03-23

**Authors:** Anthony Tokarski, Paul M Courtney, Carl Deirmengian, Stephanie Kwan, Joseph McCahon, Gregory K Deirmengian

**Affiliations:** 1 Orthopaedic Surgery, Rothman Orthopaedic Institute, Philadelphia, USA; 2 Orthopaedic Surgery, Jefferson Health New Jersey, Stratford, USA

**Keywords:** mortality rate in sepsis, hip and knee replacement, periprosthetic joint infection, total joint arthroplasty, systemic inflammatory response syndrome [sirs]

## Abstract

Introduction

Periprosthetic joint infection (PJI) is one of the most devastating complications of total joint arthroplasty. Systemic symptoms of infection may indicate a patient who is at a higher risk of serious complications. The goal of this study was to determine if systemic symptoms of infection in the setting of PJI were associated with greater in-hospital mortality.

Materials and methods

We used our institutional database to identify all patients urgently treated for deep PJI from 2002-2012. Records were reviewed to collect demographics, surgical data, vital signs prior to surgical intervention, blood and intraoperative culture results, preoperative intensive care unit (ICU) admissions, and deaths that occurred during the hospital admission. Patients were classified as having systemic inflammatory response syndrome (SIRS) based on the criteria established by the American College of Chest Physicians and the Society of Critical Care Medicine.

Results

During the 10-year timeframe of our study, 484 patients were treated emergently for deep infection, with 130 (27%) meeting SIRS criteria preoperatively and 31 (6%) of the patients with SIRS having positive blood cultures. Patients with positive blood cultures and SIRS demonstrated a higher in-hospital mortality rate (p < 0.001). Neither SIRS nor SIRS with positive blood cultures were associated with ICU admission.

Discussion

Occasionally, PJI can spread beyond the affected joint, showing physical symptoms of systemic illness and bacteremia. This study demonstrates that patients with SIRS and positive blood cultures are at an increased risk of in-hospital mortality. These patients should be monitored closely before definitive treatment in order to minimize their mortality risk.

## Introduction

As the number of total knee and hip arthroplasties performed continues to rise, the number of periprosthetic joint infections (PJI) will concomitantly increase [1]. In 2007, Kurtz et al. projected the number of total hip and knee arthroplasties will increase by 174% and 673% by 2030, respectively. The number of total hip and knee revisions is also projected to grow by 137% and 601%, respectively, between 2005 and 2030 [2]. Periprosthetic joint infection is a rare but devastating complication with an incidence of 1-2% [3-5]. This complication often negatively impacts patient outcomes due to additional hospitalizations, surgical procedures, and side effects secondary to prolonged antibiotic use. In addition, PJI may cause substantial patient morbidity and mortality. Fisman et al. estimated the 30-day mortality risk with surgical intervention for PJI to be 0.4%-1.2% for 65-year-old patients and 2%-7% for 80-year-old patients [6].

The American Academy of Orthopaedic Surgeons and the Musculoskeletal Infection Society have provided detailed guidelines for the evaluation and diagnosis of periprosthetic hip and knee infection [7,8]. Despite these specific guidelines, diagnosing PJI remains challenging. Many patients with PJI may not receive appropriate treatment until after an extensive work-up is complete, often including serological inflammatory markers, joint aspiration, advanced diagnostic imaging, and on many occasions, multiple referrals [7]. A prolonged delay in diagnosis may lead to systemic illness should localized joint infection spread diffusely, potentially requiring hospital admission and more emergent treatment. 

To date, there is a paucity of literature investigating the implications of the systemic manifestation of PJI. The purpose of this study was to determine if patients with PJI who presented with manifestations of systemic illness had an increased risk of intensive care unit (ICU) admission or in-hospital mortality.

## Materials and methods

This retrospective study was approved by the Thomas Jefferson University hospital institutional review board (IRB) (# 08R.207). Using our institutional database, we identified 1402 orthopaedic patients between 2002 and 2012 who were admitted to the hospital with a diagnosis of periprosthetic joint infection (ICD9 code 996.66). Patients who received nonsurgical care, were not treated for an infected joint replacement, or who had surgery involving only superficial irrigation and debridement were excluded from the study. Hospital and outpatient electronic medical records were used to identify which patients were either admitted through the emergency department or directly admitted from the outpatient clinic for PJI of the hip or knee.

Preoperative vital signs and laboratory values were recorded to determine which patients experienced systemic inflammatory response syndrome (SIRS) preoperatively. SIRS was defined using the criteria set by the American College of Chest Physicians and the Society of Critical Care Medicine (Table 1) [9].

**Table 1:** Systemic Inflammatory Response Syndrome (SIRS) Criteria defined by American College of Chest Physicians and the Society of Critical Care Medicine

SIRS Criteria [9]
Blood White blood cell (WBC) < 4x10^9 or > 12x10^9 B/L
Body Temperature < 96.8º or > 100.4^º ^F
Heart rate > 90 bpm
Respiration rate > 20 breaths/minute

Patients who fulfilled at least two criteria within a 24-hour period preoperatively were considered to have SIRS. Laboratory records were also used to identify patients who had positive blood cultures. Additionally, inpatient electronic medical records were used to identify which patients were admitted to the ICU as well as those who died during their admission. 

For statistical analysis, Fischer’s exact test was used for all comparisons of all categorical variables. Linear regression was used to determine if there was an association between the time from admission to surgery and in-hospital mortality.

## Results

From 2002-2012, 851 patients underwent surgical treatment of PJI. Of those, 564 were determined to have been treated on an urgent basis, with 80 undergoing superficial irrigation and debridement only, leaving 484 patients for final analysis. Of the total cases, 291 involved deep knee infections, 187 in the hip. There were also five cases of bilateral knee infections, and one case of a simultaneous hip and knee infection. Treatment modalities included placement of an antibiotic spacer (268), modular component exchange (100), irrigation and debridement (87), one-stage exchange arthroplasty (13), Girdlestone arthroplasty (11), above-the-knee amputation (three), and knee fusion (one) (Table 2).

**Table 2:** Surgical Procedures

Surgical Procedures		
Procedure	Hip	Knee
Spacer Placement	93	175
Modular Exchange	13	87
Irrigation and Debridement	60	27
One Stage Exchange	10	3
Girdlestone	11	N/A
Above Knee Amputation	N/A	3
Fusion	N/A	1

Surgery was performed 0-44 days after admission, with an average of 2.5 days after admission. After reviewing admission vital signs and laboratory values, we identified 130 (26.8%) patients with SIRS preoperatively. Zero SIRS criteria were met in 182 patients, one SIRS criterion was met in 172 cases, two SIRS criteria met in 75 cases, three SIRS criteria met in 45 cases, and four SIRS criteria met in 10 cases. Of the 55 cases of SIRS with three or four criteria there was a 11% mortality rate versus 0.6% in patients presenting with zero, one, or two SIRS criteria (p < 0.001). Additionally, the odds of in hospital mortality increased with increasing number of SIRS criteria met (OR 4.1; 95% CI: 2.1-9.6), (Table 3).

**Table 3:** Systemic Inflammatory Response Syndrome (SIRS) Criteria and In-Hospital Mortality

Number of Criteria	N	Deaths (%)
0	180	0 (0)
1	172	2 (1.2)
2	75	0 (0)
3	45	2 (4.4)
4	10	3 (30)

Tachycardia was the most common presenting SIRS criteria in our cohort. Blood cultures were drawn in 269 patients. A total of 53 of these patients grew at least one organism in one or more samples. Patients with positive bacterial cultures were more likely to present with SIRS (31/53) compared to patients with negative bacterial cultures (52/216), (p < 0.001). Additionally, patients who presented with SIRS were more likely to have blood cultures drawn (83/130) than patients who did not present with symptoms of SIRS (186/354), (p = 0.030). The mean age of patients who met SIRS criteria with a positive blood culture was 69 compared to 66 for those who did not (p = 0.59). The odds of being septic increased per point increase in Charlson Comorbidity Index (p = 0.003).

At least one positive blood culture was present in 31 (23.8%) of the patients who met SIRS criteria. The most common organism that grew from blood cultures was methicillin sensitive Staphylococcus aureus (MSSA) in 35.5% (11/31) of patients. Methicillin resistant Staphylococcus aureus (MRSA) grew in nine cases (29%), three patients experienced polymicrobial bacteremia (9.7%), two patients grew Streptococcus mitis (6.5%), one patient was positive for coagulase negative Staphylococcus, one patient grew Streptococcus intermedius, one patient was positive for Streptococcus pneumoniae, one patient grew Group B, Beta hemolytic Streptococcus, one patient was positive for Morganella morganii, and one patient grew Candida glabrata (Table 4).

**Table 4:** Organisms Grown in Blood Cultures in Systemic Inflammatory Response Syndrome (SIRS) Positive Patients

Organism	n
Methicillin sensitive Staphylococcus aureus (MSSA)	11
Methicillin resistant Staphylococcus aureus (MRSA)	9
Polymicrobial	3
Streptococcus mitis	2
Streptococcus intermedius	1
Streptococcus pneumoniae	1
Group B, Beta hemolytic Streptococcus	1
Coagulase Negative Staphylococcus	1
Morganella morganii	1
Candida glabrata	1

The overall in-hospital mortality rate was 1.4% for all patients in our cohort (7/484). Patients who met SIRS criteria and had positive blood cultures had a higher mortality rate than the remainder of the cohort (12.9% vs 0.7%; p < 0.001). Of the 53 patients who had positive blood cultures, four died during the hospital admission while two patients without positive blood cultures died (7.5% vs. 0.9%; p = 0.015). The remaining patient who died did not have blood cultures drawn. Of the 31 patients meeting SIRS criteria with positive blood cultures, four died while hospitalized on average 20 days (9-47) postoperatively. Two of these patients were females and two were male. Three patients were hospitalized for infected prosthetic hips while one patient was treated for an infected knee, and were taken to the operating room an average of 3.5 days after admission. Two were treated with antibiotic spacers, one with an irrigation and debridement, and one with an exchange of modular components. In comparison, the 27/31 patients who had SIRS and positive blood cultures who did not die while hospitalized were taken to the operating room an average of 2.81 days after admission. The median time to surgery of those patients who died was four days (range 0-15) compared to a median time to surgery of one day for patients who did not die (range 0-44). This difference trended towards statistical significance (OR 1.08, p = 0.061).

In total, 242 patients were placed on preoperative antibiotics (defined as a minimum of one day preoperatively) and 242 patients did not have preoperative antibiotics. One patient in the group with no preoperative antibiotics died while in the hospital (0.4%) while six patients in the group treated with antibiotics died (2.5%). While this did not reach statistical significance (p = 0.12), this study was underpowered to study this comparison. A power analysis revealed that 549 patients per group would have been required.

Of the patients treated with antibiotics, 99 received multiple antibiotics, 96 were treated with an antibiotic with MRSA coverage, 30 were treated with an antibiotic that covered gram negative organisms, 16 were treated with a cephalosporin, and one was treated with an antibiotic with anaerobic coverage. Five (5.1%) patients treated with multiple antibiotics died during the hospital stay and one (6.3%) patient treated with a cephalosporin died.

The effect of spectrum of coverage on mortality did not reach statistical significance (p = 0.08). Of the patients that died with SIRS and positive blood cultures, one had synovial and blood cultures positive for both Proteus mirabalis and MRSA. One patient grew MSSA in joint tissue and blood cultures. Another patient grew MSSA and coryneform species in tissue cultures and MSSA in blood cultures. The final patient had polymicrobial growth from tissue cultures (MSSA, coagulase negative Staphylococcus, coryneform species, and Enterococcus faecalis) and MSSA in blood cultures. The cause of death for these four patients was documented as organ failure from septic shock in three cases and pulmonary embolism in the final case. In addition, three other patients in the cohort died, with one having SIRS but negative blood cultures. Two patients were treated for PJI of the hip and one was treated for PJI of the knee.

In this series, 27 patients were admitted to the intensive care unit (ICU) postoperatively, 15 of which were being treated for PJI of the hip and 12 for PJI of the knee. Four of these patients died during their hospital stay. Additionally, three patients were admitted to the ICU preoperatively and did not die during hospitalization. Of the patients who presented with SIRS, eight (6.1%) were admitted to the ICU postoperatively. This is in comparison to the 19 (5.4%) patients who did not present with SIRS and were admitted to the ICU. This difference was not statistically significant (p = 0.662). Three of the 31 patients with SIRS and positive blood cultures were admitted to the ICU postoperatively compared to 24 of the 453 patients who did not have SIRS and positive cultures (p = 0.230). Patients who were admitted to the ICU were more likely to die during their hospital stay than patients who were not admitted to the ICU (14.8% vs. 0.7%; p <0.001).

## Discussion

To our knowledge, this study is the first to examine the correlation between systemic illness with periprosthetic joint infections and mortality risk. Several authors have identified PJI as a risk factor for increased mortality. Zmitowski et al. found a significant increase in mortality in patients undergoing surgical intervention for PJI compared with patients undergoing aseptic revision arthroplasty [10]. They showed that the mortality risk is greatest between 30 days and one year postoperatively. Significant predictors of one-year mortality in patients with a PJI included advanced age, increased Charlson Comorbidity Index, cardiac disease, and history of cerebrovascular accident. Berend et al. reported the 90-day mortality rate after a two stage exchange to be 4%, with an additional 7% of patients dying before they were able to undergo the second stage [11].

In this cohort, patients who presented with minimal (two or fewer) or no SIRS did not have a statistically significantly higher mortality rate than patients who did not show systemic symptoms of illness. However, patients who presented with three or four SIRS criteria did have a significantly higher mortality rate than patients with two or fewer SIRS criteria. This may suggest that patients who exhibit more severe signs of systemic disease may be at a higher risk of death and should be monitored closely while awaiting definitive treatment.

An important finding of our study was that patients that presented with SIRS and positive blood cultures had an increased in-hospital mortality rate that has not been identified in prior studies. Of note, the three patients who died after presenting with preoperative SIRS and positive blood cultures were hospitalized for twice as long (4.71 vs 2.81 days) prior to their surgery than those who did not die postoperatively. Although we were unable to show a statistically significant correlation between the number of preoperative hospital days and mortality, this finding certainly warrants further investigation. This insight may be important for future studies, as it is a variable that may be controlled by healthcare providers, unlike the mortality predictors reported by Zmitowski et al., in which patient host factors predominated [10].

The results of our study suggest that patients presenting to the hospital with a PJI should be closely monitored for signs of systemic illness. Our results show that SIRS criteria and blood culture results are useful tools to achieve this goal. Patients with a systemic illness are often immunologically compromised and may be unable to effectively mount an appropriate host response. In studies by Cierny et al. and McPherson et al., patients who were medically ill were more likely to die from a PJI [12,13]. The results of this study support the concept that patients whose PJI manifests as systemic illness are at an increased risk of mortality.

In our study, systemic illness was not identified as a risk factor for ICU admission postoperatively. In fact, the rate of ICU admission was very similar (6.2% vs. 5.4%) regardless of preoperative systemic symptoms. This finding may be secondary to the fact that these patients experiencing perioperative complications that were unrelated to their PJI that required transfer to the ICU. In a retrospective study by Chang et al., revision total hip arthroplasty and debridement for an infected total hip arthroplasty carried a high risk of postoperative complications that required transfer to the ICU [14].

We recognize several limitations to our study. Due to this study’s retrospective nature, we were unable to standardize the treatment of patients that presented with PJI. Additionally, since this study spans a 10-year period, diagnostic criteria and management protocols may have changed over the study period. Lastly, because of the retrospective design of our study and the relatively low number of patients included, we were unable to control for other potentially confounding factors between the patient cohorts. It is certainly possible that the patients who met SIRS criteria had different demographics and a profile of medical comorbidities than those who did not meet SIRS criteria, which could have factored into the increased mortality found in our results. Thus, the relationship between meeting SIRS criteria and increased mortality should only be considered a correlation. Nonetheless, treating physicians may find the correlation important in guiding decisions for such patients.

## Conclusions

Our study demonstrates that patients with manifestation of systemic illness in the setting of PJI are at an increased risk of in-hospital mortality. Patients presenting to the hospital with a PJI should be monitored closely for signs of systemic disease with consideration for urgent surgical management should these symptoms present.
